# Dietary fiber content influences small intestinal histomorphology and cecal microbial composition of laying hens

**DOI:** 10.1016/j.psj.2026.107292

**Published:** 2026-06-13

**Authors:** S. Das, I. Halle, T.M. Fuchs, A. Dudde, E.T. Krause, A. Patt

**Affiliations:** a Institute of Animal Welfare and Animal Husbandry, Friedrich-Loeffler-Institute, Celle, Germany; b Albrecht Daniel Thaer-Institute of Agricultural and Horticultural Sciences, Faculty of Life Sciences, Humboldt-Universität zu Berlin, Berlin, Germany; c Institute of Animal Nutrition, Friedrich-Loeffler-Institute, Brunswick, Germany; d Institute of Molecular Pathogenesis, Friedrich-Loeffler-Institute, Jena, Germany

**Keywords:** Insoluble fiber, Intestinal morphology, Nutrient absorption, Gut microbial diversity, Chicken

## Abstract

This study investigated the effect of increasing proportions (3%, 6%, 9%) of insoluble dietary fiber on small intestinal histomorphology and cecal microbial composition of Lohmann Tradition hens. At 21 weeks of age, 12 groups of 20 hens each were assigned to the three feeding treatments. The three diets were isocaloric and therefore differed in the proportions of main energy sources e.g. dietary fat. In week 65, three hens from each group were sacrificed to collect samples from the small intestine (duodenum, jejunum, ileum), and luminal and mucosal samples from the cecum. Villus height, villus width, and crypt depth were measured in the three small intestinal sections. Villus surface area and villus height-crypt depth ratio were calculated. Villus height and villus surface area increased with dietary fiber content in all three sections, indicating a potential increase in nutrient absorption in the small intestine with increasing fiber. With regards to cecal microbial composition, 16S rRNA gene amplicon sequencing was performed with the luminal and the mucosal samples to assess microbial diversity and relative abundances of bacterial genera. Our results showed that increasing fiber content increased the luminal alpha-diversity and shifted the microbial community structure at the genus level in both lumen and mucosa. A principal component analysis (PCA) of genus-level relative abundance data indicated that increasing fiber content was associated with an increased luminal abundance of butyrate-associated bacteria. In the mucosa, higher fiber content was associated with an increased abundance of mucosa-associated bacteria. Overall, insoluble fiber feeding of up to 9% was associated with beneficial changes in the small intestinal histomorphology and the cecal microbial composition, suggesting potential benefits for gut health. Despite the higher fat content in the 6% and 9% fiber diets, the increased fiber levels may have mitigated negative effects on gut health usually observed with high dietary fat.

## Introduction

Dietary fiber is an important component of the diet of monogastric animals e.g. poultry, as it positively contributes to gut functionality, in particular by gastro-intestinal development, maintenance of intestinal physiological roles, and modulation of the gut microbial composition (Jha et al., 2019; Jha and Mishra, 2021). Dietary fibers constitute a heterogeneous group of carbohydrate polymers that are resistant to digestion and absorption in the small intestine, but are partially or nearly completely fermented by the microbial community in the large intestine. Dietary fibers exhibit different functional properties based on their characteristics, such as solubility, viscosity, and fermentability (Mudgil and Barak, 2019).

Soluble fibers have largely been known for their anti-nutritive properties by producing high viscosity in the small intestine and thereby inhibiting digestion and absorption of nutrients, which could potentially compromise bird performance (Jha and Mishra, 2021). Insoluble fibers have been traditionally regarded as inert nutrient diluents with little or no nutritive value in poultry diets (Hetland et al., 2004). However, recent findings suggest that insoluble fiber feeding improved intestinal morphology for example in poultry, characterized by increased villus height and villus surface area. For instance, a diet with 4% crude fiber supplemented with lignocellulose increased the ileal villus height of broilers along with an increase in growth performance traits such as body weight and feed conversion ratio, compared to a non-supplemented diet with 3.4% crude fiber (Makivic et al., 2019). As nutrient absorption occurs through the villus surface, Makivic et al. (2019) proposed that the observed morphological improvements in the ileum might have increased nutrient absorption in the small intestine of the birds, thereby potentially improving performance traits. In laying hens, diets exhibiting 3 to 4% crude fiber and supplemented with varying insoluble fiber sources (e.g. sunflower meal, wheat bran) increased villus height and villus surface area in the small intestine, as compared to a non-supplemented diet (Abdollahi et al., 2021; Koçer et al., 2021). However, in contrast to each other, Koçer et al. (2021) reported a simultaneous increase in laying performance with insoluble fiber supplementation, whereas Abdollahi et al. (2021) stated that laying performance was not affected by insoluble fiber supplementation in their study.

Soluble and insoluble fibers have also been traditionally thought to differ in terms of fermentability, with soluble fibers being extensively fermentable by the gut microbes in the large intestine, and insoluble fibers considered nearly non-fermentable (Jha et al., 2019). However, no major differences were found in the in-vitro fermentability (using pig fecal microbiota) of soluble and insoluble fiber fractions from three different refined flour (wheat, rye, and barley) substrates (Comino et al., 2018). Instead, fermentability of insoluble fiber was seen to differ with the source of fiber (i.e. flour type) indicating that fermentability is more strongly affected by the chemical composition of fiber rather than its solubility. Other studies in pigs and humans reported that different insoluble fibers may be fermented to varying extents by the gut microbes (Williams et al., 2019). This suggests that insoluble fiber supplementation in the diet is likely to affect the gut microbial composition. Consistent with this, recent studies in poultry have reported an increased microbial diversity or an increase in the relative abundances of bacteria involved in the production of short-chain fatty acids (**SCFA** e.g. acetate, propionate, butyrate) or both owing to insoluble fiber supplementation in the diet. For instance, diets containing 2% and 4% lignocellulose increased the cecal microbial diversity and the relative abundances of butyrate-producing bacteria such as *Faecalibacterium* (Eubacteriales: Oscillospiraceae) in dual purpose chickens (Hou et al., 2020). Another study with broilers showed that 1% cellulose supplementation in the diet increased the relative cecal abundance of the cellulolytic bacterial genus *Alistipes* (Bacteroidales: Rikenellaceae) that might stimulate propionate synthesis (De Maesschalck et al., 2019). These SCFA, especially butyrate, have been reported to provide energy to intestinal epithelial cells, regulate intestinal barrier function, and mediate immune responses, for example by upregulating anti-inflammatory cytokines (Liu et al., 2021; Martin-Gallausiaux et al., 2021). Therefore, insoluble fiber in the diet might positively alter the gut microbial composition in poultry.

Poultry have been seen to voluntarily consume litter materials, such as wood shavings or straw, which are often high in fiber (Hetland et al., 2003; 2004). Laying hens fed low fiber diets seemed to compensate for fiber deficit by consuming higher amounts of wood shavings and free feathers from the litter (Patt et al., 2022). However, research on the potential health-promoting effects of insoluble fiber feeding, especially in terms of microbiota composition, is limited in poultry compared to other monogastric animals like pigs. Moreover, most of the studies examine the microbial composition only in the lumen and not the mucosa of the cecum, for instance. The mucosa is coated by a protective gel called mucus, which is majorly composed of mucin glycoproteins. Metabolic specialists among the microbial community such as *Bacteroides* spp. (Bacteroidales: Bacteroidaceae) and *Akkermansia muciniphila* (Verrucomicrobiales: Akkermansiaceae) are able to degrade the mucin glycoproteins, and can therefore selectively colonize the mucosa (humans: Raimondi et al., 2021). Previous studies with broilers have shown that luminal microbiota composition may differ from the mucosal composition of the cecum (Olsen et al., 2008; Awad et al., 2016). Hence, it is important to identify microbiota composition in both the lumen and the mucosa within a gut section.

The research described herein is a part of a larger study that investigated the effects of three diets with linearly increasing proportions of targeted insoluble dietary fiber (3%, 6%, 9% of wheat straw), but with similar amounts of metabolizable energy, in laying hens. Data collected from this study on behavior (feather pecking, aggressive pecking, and locomotor activity), plumage quality, gizzard content, and performance traits (laying performance, body weight, and estimated feed conversion rate) were published previously (Patt et al., 2022). The current article presents data collected from the same study on small intestinal histomorphology and cecal microbial composition in both lumen and mucosa. Wheat straw is comprised of 34 to 40% cellulose, 30 to 35% hemicellulose and 14 to 15% lignin, all of which are classified as insoluble fiber (Sun et al., 1995; 1998). Here, we tested the hypothesis that higher levels of insoluble fiber in the diet might improve intestinal histomorphology and microbial composition.

## Materials and methods

### Ethics statement

The experiment was carried out between March 2017 and March 2018 at the Friedrich-Loeffler-Institut in Celle, Germany. It was performed in accordance with the German Animal Protection Law and was approved by the German Lower Saxony State Office for Consumer Protection and Food Safety (No. 33.9–42 502-04-17/2488).

### Animals and housing

A total of 240 Lohmann Tradition hens (18-weeks-old) were obtained from a commercial breeder and randomly distributed into 12 groups, each group comprising 20 hens. Each group was housed in a 4 m^2^ (2 × 2 m) pen with a 2.4 m^2^ (2.0 × 1.2 m) elevated slatted area, a group nest (1.0 × 0.4 × 0.7 m, littered with buckwheat spelts), a 1.6 m^2^ (2.0 × 0.8 m) littered (wood shavings) area, and multiple perches. Fresh litter was added as required. A standard 14:10 h light:dark cycle (light period: 4 a.m. to 6 p.m.) was used. Hens were offered food and water ad-libitum that were accessible from the elevated area. Upon arrival, the hens were fed the same standard commercial laying hen feed (ME 2677 kcal/kg i.e. 11.2 MJ/kg, dry matter 88.66%, crude protein 15.5%, crude fiber 3.4%) until 20-weeks-old. At 21-weeks-old, the 12 groups were allocated to three different feeding treatments, each treatment comprising of four replicates (therefore 80 hens per treatment). At 65-weeks-old, three hens from each of the 12 groups (total 36 hens) were sacrificed to collect histomorphological samples of the small intestine (duodenum, jejunum, ileum), and intestinal contents of the cecum to assess microbial composition. During sacrifice, hens were individually stunned with an electric stunner and subsequently bled by a ventral neck cut opening both carotid arteries and jugular veins.

### Feeding treatments

The three different experimental diets varied in dietary fiber content, containing either 3%, 6% or 9% fiber. Each group of hens received one of the three diets starting from 21-weeks-old until 65-weeks-old. The three experimental diets were isocaloric (ME 2701 kcal/kg i.e. 11.3 MJ/kg), but differed in crude fiber and neutral detergent fiber content, achieved by the addition of wheat straw (Table 1). Feed was pelleted to ensure that the ground wheat straw was evenly distributed in the feed and consumed by the hens. Dry matter, crude protein, ether extract, crude fiber, neutral detergent fiber, and ash content were determined according to methods of the Verband Deutscher Landwirtschaftlicher Untersuchungs- und Forschungsanstalten (VDLUFA, 2012). Standard commercial laying hen diets typically contain approximately 2-3% crude fiber (Desbruslais et al., 2021). Moreover, poultry diets are commonly cereal based, with carbohydrates occurring predominantly as readily digestible starch (National Research Council, 1994). Accordingly, starch provided approximately 58% of the metabolizable energy in the 3% fiber diet (Table 1). The 3% dietary fiber diet was therefore used as an experimental control. With increasing dietary fiber content, the dietary energy source was increasingly shifted from carbohydrates to fat to maintain isocaloric composition.

**Table 1:** Ingredient composition (g/kg) of the three experimental diets, fed to Lohmann Tradition hens, that differed in the amount of dietary fiber (3%, 6%, 9%) but not in metabolizable energy.

Feed ingredient	Dietary fiber content		
	3%	6%	9%
Corn	302.8	125.8	128.2
Wheat	300.0	300.0	120.0
Wheat straw	10.0	66.6	147.7
Oat	50.0	70.0	80.0
Soya bean meal	193.7	294.3	338.5
Soya oil	30.0	30.0	73.5
Premix^1^	10.0	10.0	10.0
Calcium carbonate	85.7	84.8	82.8
Dicalcium phosphate	12.8	13.3	13.8
Sodium chloride	3.6	3.5	3.2
DL-methionine	1.4	1.7	2.3
Dry matter^2^	891.5	896.4	916.1
Crude protein^2^	156.8	160.7	173.3
Crude fiber^2^	25.3	65.7	83.9
Neutral detergent fiber^2^	89.9	152.7	178.6
Crude fat^2^	63.2	104.2	149.4
Crude starch^2^	451.1	330.6	232.8
ME (kcal/kg)^3^	2701	2701	2701
Calcium^4^	37.5	37.5	37
Phosphorus^4^	5.4	5.4	5.3
Lysine^4^	7.7	7.7	7.7
Methionine + Cystine^4^	6.9	6.9	6.9

1 Vitamin–mineral premix provided per kilogram of diet: Iron, 40 mg; Copper, 10 mg; Zinc, 80 mg; Manganese, 100 mg; Selenium, 0.25 mg; Iodine, 1.2 mg; Cobalt, 0.21 mg; Vitamin A (retinyl acetate), 10,000 IU; Vitamin D3, 2,500 IU; Vitamin E (dl-α-tocopheryl acetate), 20 IU; Vitamin K3, 4 IU; Thiamine, 2.5 mg; Riboflavin, 7 mg; Pyridoxine, 4 mg; Vitamin B12, 20 µg; Nicotinic acid, 40 mg; Pantothenic acid, 10 mg; Folic acid, 0.6 mg; Biotin, 25 µg; Choline chloride, 400 mg.

2 Analyzed values.

3 Calculated values according to WPSA (1985): AMEn (kJ/g) = 15.51 × crude protein + 34.31 × crude lipid + 16.69 × starch + 13.01 × sugar (Vogt, 1986).

4 Calculated values.

### Data collection

**Histomorphology of the Small Intestine:** Immediately following sacrifice, the abdominal cavity was opened and the entire gastrointestinal tract was removed. The small intestine was isolated and segments of approximately 1 to 2 cm were taken from the midpoints of each duodenum, jejunum, and ileum. Segments were fixed in 4% formalin solution for 2 to 3 days. Next, the fixed tissue samples were dehydrated in increasing concentrations of ethyl alcohol, cleared in xylol, and embedded in paraplast (Paraplast Plus, Art. Nr. X881.1, Carl Roth GmbH + Co. KG, Karlsruhe, Germany). The embedded samples were then cooled at −20°C for approximately 1 hour and cut as 4 μm thick sections with a microtome blade (model N35HR, FEATHER Safety Razor Co., Ltd., Osaka, Japan), mounted onto clean glass slides, and stained with hematoxylin and eosin (H&E staining).

The stained samples were photographed under a microscope and at least five (and up to ten) intact, well-oriented intestinal villus-crypt units from each intestinal section of each hen were used for morphometric measurements. The criterion for villus selection was based on the presence of intact lamina propria. Villus height was measured as a straight line from the tip of the villus to the base (i.e. until the villus-crypt junction). Villus width was measured halfway along the length of the villus, 90° to longitudinal direction. Crypt depth was measured as the depth of the invagination between two consecutive villi. These measurements were carried out using the image-analysis software AxioVision (Carl Zeiss Microscopy GmbH, Jena, Germany). Villus surface area (= villus height × villus width) and the ratio of villus height and crypt depth (**VH:CD**) also were calculated.

**Cecal microbial composition:** Following sacrifice of the hens, their ceca were isolated. The luminal contents of one cecum were collected with a spatula and placed inside a 2 ml sterile microcentrifuge tube containing 600 µl of stool stabilizer (Catalog No. 1038111100, Invitek Molecular GmbH, Berlin, Germany). The contents of the tube were vortexed thoroughly and placed immediately on dry ice, and finally stored at −80°C. The other cecum was cut longitudinally and clamped onto a cardboard with pins (mesenteric side of the cecum resting on cardboard) with the cardboard standing upright in a rinsing vessel. The cecal contents were washed thoroughly with sterile phosphate-buffered saline (PBS) following which a scraping was performed along the mucosa with a new sterile scalpel blade. The collected mucosal sample was placed inside a sterile microcentrifuge tube and placed immediately on dry ice, and finally stored at −80°C.

The cecal samples (72 samples in total i.e. two samples from each hen) were analyzed at the Core Facility Microbiome, ZIEL Institute for Food & Health, Technical University of Munich, Freising, Germany. DNA extraction and 16S rRNA gene amplicon sequencing was performed as described in Lagkouvardos et al. (2015). Briefly, microbial cells were mechanically lysed using a bead-beater (Catalog No. 6004500, FastPrep-24, MP Biomedicals GmbH, Eschwege, Germany). DNA was purified on NucleoSpin gDNA columns (Item No. 740230.50, Macherey-Nagel GmbH & Co. KG, Düren, Germany). PCR was performed in duplicates, and the V3-V4 region of 16S rRNA genes was amplified in a two-step approach using primers 341F and 785R. PCR products were purified with magnetic beads (AMPure XP beads, Beckman Coulter GmbH, Krefeld, Germany), pooled in equimolar ratios and sequenced in paired-end mode on Illumina MiSeq machines (Illumina GmbH, Berlin, Germany).

### Data processing and statistics

**Histomorphology of the Small Intestine:** Statistical analysis was conducted in R version 4.3.1 (R Core Team, 2023). The influence of the fixed effects (feeding treatment and intestinal section) and their interaction on the outcome variables (listed below) was analyzed using Bayesian linear mixed-effects models following the *blmer* method from the blme package (Chung et al., 2013). The following outcome variables were analyzed: villus height (µm), villus surface area (mm^2^), crypt depth (µm), and VH:CD (Table 2). Data for the continuous fixed effect, i.e. feeding treatment (3%, 6%, 9% dietary fiber), were normalized, and sum contrasts were used for the factor variable, i.e. intestinal section (duodenum, jejunum, ileum), such that P-values of main effects remained meaningful even in the presence of an interaction. Hen identity (henID) nested in pen identity (penID) were considered as random effects for the models. A maximum model (including fixed effects as main effects and their interaction) was set up and compared with a minimal model (only including an intercept) as well as with models that each included a fixed effect or their interaction (i.e. investigating the effect of the interaction and each fixed effect separately). Model comparisons were done using parametric bootstrap (package pbkrtest, Halekoh and Højsgaard, 2014). Model assumptions were verified using graphical analysis of residuals (package DHARMa, Hartig, 2024), focusing on normality of errors and random effects as well as homogeneity of variances. All outcome variables were log-transformed, to meet model assumptions. Model predictions were generated via parametric bootstrap (n = 1000) using the *bootMer* function. Confidence intervals (95%) were calculated using the boot package (Davison and Hinkley, 1997; Canty and Ripley, 2025). When reporting results, we focused on the interaction term if it was statistically significant and described how the interaction pattern may be interpreted. If the interaction term was statistically non-significant, we reported the interpretation of the main effects of feeding treatment and intestinal section.

**Table 2:** Test statistics and P-values of the Bayesian linear mixed-effects models for small intestinal histomorphology of Lohmann Tradition hens fed three experimental diets that differed in the amount of dietary fiber (3%, 6%, 9%) but not in metabolizable energy.

Fixed effect	Degrees of freedom (df)	$\chi_{df}^{2}$	P-value
Villus height			
All (maximum model vs. intercept only model)	5	1337.92	< 0.001
Feeding treatment × intestinal section	2	19.62	< 0.001
Feeding treatment	1	10.42	0.002
Intestinal section	2	481.20	< 0.001
Villus surface area			
All (maximum model vs. intercept only model)	5	696.28	< 0.001
Feeding treatment × intestinal section	2	20.13	< 0.001
Feeding treatment	1	3.67	0.081
Intestinal section	2	185.89	< 0.001
Crypt depth			
All (maximum model vs. intercept only model)	5	63.51	< 0.001
Feeding treatment × intestinal section	2	6.62	0.039
Feeding treatment	1	5.47	0.022
Intestinal section	2	25.91	< 0.001
Villus height-crypt depth ratio (VH:CD)			
All (maximum model vs. intercept only model)	5	269.30	< 0.001
Feeding treatment × intestinal section	2	3.54	0.178
Feeding treatment	1	0.21	0.643
Intestinal section	2	27.76	< 0.001

### Cecal microbial composition

**Post-sequencing Data Processing:** The 16S rRNA gene amplicon sequencing data from the luminal and the mucosal samples were further processed and analyzed separately. The sequencing data was first processed with the Integrated Microbial Next Generation Sequencing (IMNGS) pipeline (Lagkouvardos et al., 2016) based on the UPARSE approach (Edgar, 2013). The raw read files were demultiplexed allowing up to two mismatches in barcode sequences. Paired-end reads were merged and subjected to quality filtering using USEARCH version 8.0 (Edgar, 2010). All reads were trimmed to the position of the first base with a minimum quality score of three, and only those with lengths between 350 and 500 nucleotides were retained. Sequences with more than three expected errors were further discarded, and the remaining sequences were trimmed by five nucleotides at both the forward and the reverse ends. Sequences were dereplicated for each sample and filtered for chimeras using UCHIME (Edgar et al., 2011). Filtered sequences from all samples were then pooled, sorted by relative abundance, and clustered *de novo* into operational taxonomic units (**OTU**) at 97% sequence identity. A representative sequence was selected for each OTU. Finally, all original sequences were mapped back to the representative OTU sequences resulting in one OTU table containing relative abundance counts for all samples. All OTU with a relative abundance below 0.5% in all samples were removed to prevent the analysis of spurious OTU. Taxonomic classification (up to genus level) was then assigned to each OTU using the RDP classifier version 2.11 (Wang et al., 2007). Sequence alignment was performed using MUSCLE (Edgar, 2004) and phylogenetic trees were constructed with FastTree (Price et al., 2010).

Downstream analysis of OTU tables (one for luminal and one for mucosal samples) was performed in R, following the Rhea pipeline (Lagkouvardos et al., 2017). Firstly, the OTU tables were rarefied to the minimum number of sequences observed in any sample, in order to account for differences in sequencing depth between samples. The effective Simpson diversity was used as the measure of within-sample (alpha) diversity, as effective diversities are linear measures and therefore easier to interpret than their corresponding raw indices (Jost, 2006; 2007). To estimate the relative abundances of individual taxa, taxonomic binning was performed at every taxonomic level. We kept the default settings of the Rhea pipeline to consider taxonomic variables to be effectively present with a minimum relative abundance of 0.5. Variables less than this cut-off were zeroed. Further, taxa with a median relative abundance of < 1% in all groups and ones that are not detectable in at least 30% of all samples in each group (= minimum prevalence) were excluded. We used the microbial diversity (i.e. Simpson effective index) and the relative abundances of bacterial genera for further analysis. While reporting the names of individual genera, the taxonomic placement [Order: Family] of the genus is presented.

**Principal Component Analysis:** Following taxonomic binning using the Rhea pipeline, a principal component analysis (**PCA**) was performed to reduce the dimensionality and identify major patterns in the relative abundances of bacterial genera. PCA was applied to both the luminal and the mucosal data in R using the *prcomp* function. In this context, a PCA helped to summarize overall similarities and differences in the relative abundances by grouping the variation in the data into so-called principal components (**PC**). Each PC represented a distinct pattern of variation across samples, with PC1 capturing the greatest amount of variation, followed by PC2, and so on. The loading of each genus on a PC indicated how strongly and in which direction (positive or negative) it contributed to the given PC. Higher absolute values meant that the genus had a strong influence in shaping the underlying pattern. Positive and negative loadings respectively indicated that the relative abundance of the genus increased and decreased with the PC score. Because relative abundance data are compositional in nature, centered log-ratio (**CLR**) transformation was applied to the data prior to running the PCA (Gloor et al., 2017; Quinn et al., 2018) using the microbiome package (Lahti and Shetty, 2017). Because this transformation does not work for zero values, zeros were replaced by adding a set value of 1 to all the abundance counts. After running the PCA, a scree plot of eigenvalues was examined to determine how many PC were most strongly indicated (Supplementary Figures 1 & 2). The loadings of all genera are presented in Supplementary Tables 1 & 2, and a threshold of ± 0.25 was applied to interpret patterns within a PC. For the luminal samples, orthogonal rotation of each PC was performed. Additionally, PCA biplots are presented to visually illustrate sample clustering and the alignment of genera with group separation.

**Bayesian linear mixed-effects models:** Following this, models were run to analyze the influence of the fixed effect (i.e. feeding treatment) on the Simpson effective index and the relevant PC (Table 3). Data on feeding treatment were normalized, and pen identity (penID) was used as the random effect. A model was set up with feeding treatment as the main effect and compared with an intercept-only model. Model comparisons were done and model assumptions were verified as described previously. No major deviations from the assumptions were observed. Model predictions and confidence intervals were estimated as described previously.

**Table 3:** Test statistics and P-values of the Bayesian linear mixed-effects models for cecal microbial composition of Lohmann Tradition hens fed three experimental diets that differed in the amount of dietary fiber (3%, 6%, 9%) but not in metabolizable energy.

Location	Outcome variable	Fixed effect	Degrees of freedom (df)	$\chi_{df}^{2}$	P-value
Lumen	Simpson effective index	Feeding treatment	1	8.88	0.005
	Propionate and acetate-associated bacteria	Feeding treatment	1	0.63	0.48
	Butyrate-associated bacteria	Feeding treatment	1	15.17	< 0.001
Mucosa	Simpson effective index	Feeding treatment	1	0.11	0.742
	Mucosa-associated bacteria	Feeding treatment	1	10.75	0.002
	Butyrate-associated bacteria	Feeding treatment	1	1.51	0.256

## Results

### Insoluble fiber increased villus height, villus surface area, and crypt depth in the small intestine

**Fig. 1:**
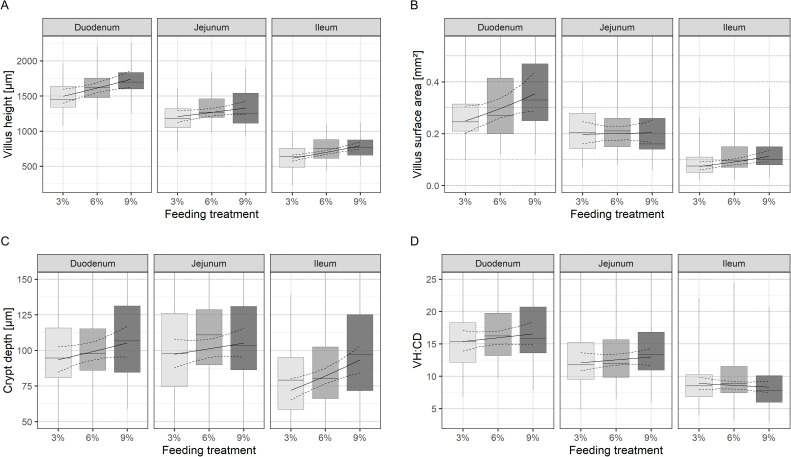
Small intestinal histomorphology of Lohmann Tradition hens fed three experimental diets that different in the amount of dietary fiber (3%, 6%, 9%) but not in metabolizable energy: (A) Villus height, (B) villus surface area, (C) crypt depth, and (D) villus height-crypt depth ratio (VH:CD), as an outcome of the interaction between feeding treatment and intestinal section (duodenum, jejunum, ileum), are presented as box-and-whiskers plots. Each box represents the interquartile range, the thick line within the box indicates the median and the whiskers represent the range of the data. The solid lines indicate the model estimates and the dotted lines represent 95% confidence intervals. All figures show the raw data for the interaction between feeding treatment and intestinal section.

Small intestinal histomorphology (villus height, villus surface area, crypt depth, and VH:CD) was assessed in the duodenum, the jejunum, and the ileum. With respect to villus height, the interaction between feeding treatment and intestinal section was statistically supported (P = 0.001, Table 2; Fig. 1A). Villus height increased with increasing dietary fiber content in all three intestinal sections, with a slightly stronger increase in the duodenum, followed by the ileum and the jejunum. Overall, villus height decreased along the small intestine from the duodenum along the jejunum to the ileum. For villus surface area, the interaction between feeding treatment and intestinal section was also statistically supported (P = 0.001, Table 2; Fig. 1B). With increasing dietary fiber content, villus surface area increased in the duodenum and, to a lesser extent in the ileum, but appeared to remain unchanged in the jejunum. Overall, villus surface area decreased from the duodenum to the jejunum to the ileum. The interaction between feeding treatment and intestinal section for crypt depth was statistically supported (P = 0.039, Table 2; Fig. 1C) showing that crypt depth increased with increasing dietary fiber content in all three intestinal sections, with a stronger increase in the ileum than in the duodenum and the jejunum. Overall, crypt depth was deeper in both the duodenum and the jejunum compared to the ileum. For VH:CD, the interaction between feeding treatment and intestinal section was not statistically supported (P = 0.178, Table 2; Fig. 1D), and VH:CD did not change with increasing dietary fiber content (feeding treatment: P = 0.643). However, VH:CD decreased from the duodenum to the ileum (intestinal section: P = 0.001).

### Insoluble fiber increased luminal microbial diversity and altered community structure

**Fig. 2:**
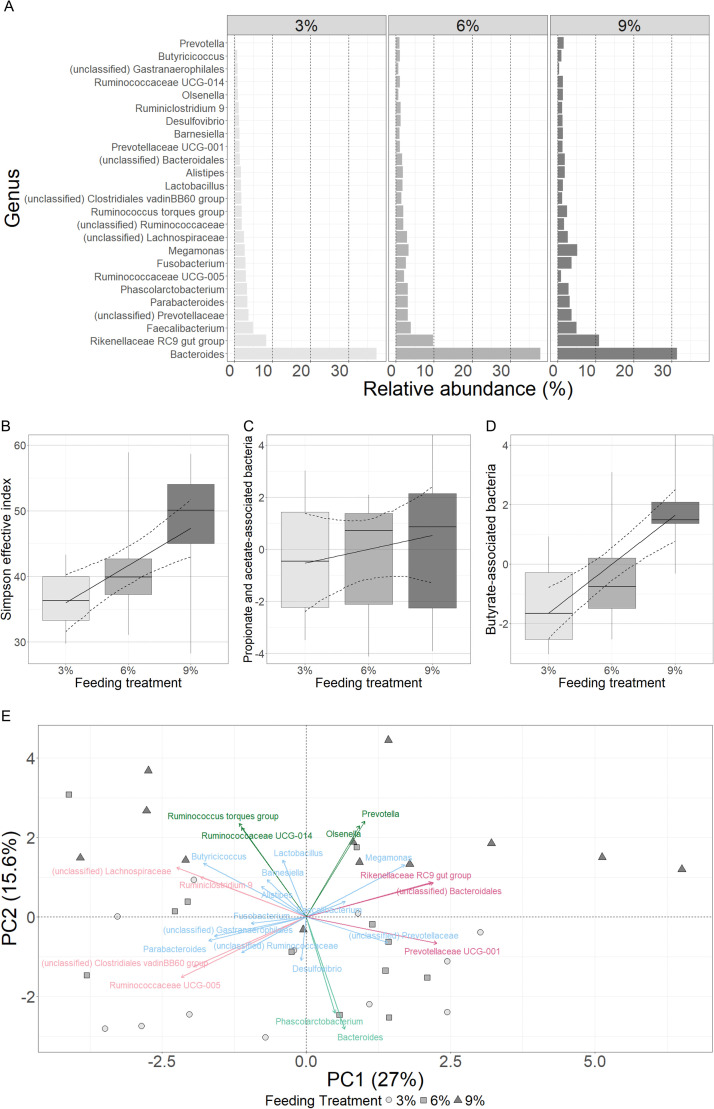
Microbial composition of the cecal lumen of Lohmann Tradition hens fed three experimental diets that different in the amount of dietary fiber (3%, 6%, 9%) but not in metabolizable energy: (A) Relative abundance (%) of bacterial genera are presented as bar plots. Each bar represents the mean relative abundance of the respective genus for each feeding treatment. (B) Microbial diversity (Simpson effective index), (C) propionate and acetate-associated bacteria, and (D) butyrate-associated bacteria, as an outcome of feeding treatment, are presented as box-and-whiskers plots. Each box represents the interquartile range, the thick line within the box indicates the median and the whiskers represent the range of the data. The solid lines indicate the model estimates and the dotted lines represent 95% confidence intervals. (E) Principal component analysis (PCA) biplot of CLR-transformed genus-level microbial relative abundance. Points represent individual samples by feeding treatment (circle: 3%; square: 6%; triangle: 9% dietary fiber), while arrows represent genera contributing to variation among samples. The first two principal components (PC) explained 27% and 15.6% of total variance respectively. The genera that loaded (loading threshold = ± 0.25) positively and negatively on PC1 are colored dark pink and light pink respectively; genera that loaded positively and negatively on PC2 are colored dark green and light green respectively; genera that did not load on either PC are colored blue.

Cecal microbial composition (microbial diversity and relative abundances of bacterial genera) was assessed in both the lumen and the mucosa. 16S rRNA gene amplicon sequencing revealed that the bacterial community in the lumen was dominated by the phylum Bacteroidetes followed by Firmicutes, with ∼60% of all genera belonging to Bacteroidetes and ∼30% to Firmicutes for each of the three feeding treatments. A total of 25 different bacterial genera were observed across all luminal samples (Fig. 2A).

The Simpson effective index (= measure of microbial diversity) increased with increasing dietary fiber content in the lumen (feeding treatment: P = 0.005, Table 3; Fig. 2B).

A PCA was performed to reduce dimensionality. The first two PC were most strongly indicated by the PCA (Supplementary Figure 1). PC1 (27% of variance explained) was associated with positive loadings of genera previously described as propionate and acetate producing (Prevotellaceae UCG-001, Rikenellaceae RC9 gut group, and an unclassified genus from the order Bacteroidales) and negative loadings of genera reported to be linked to butyrate production (unclassified Lachnospiraceae, Ruminococcaceae UCG-005, unclassified genus of the Clostridiales vadin BB60 family, and *Ruminiclostridium* 9 [Eubacteriales: Oscillospiraceae]). PC1 was therefore referred to as the ‘propionate and acetate-associated bacteria’. PC2 (15.6% of variance explained) was associated with positive loadings of genera reported to be directly or indirectly linked to butyrate production (Ruminococcaceae UCG-014, *Prevotella* [Bacteroidales: Prevotellaceae], members of the *Ruminococcus torques* group, and *Olsenella* [Coriobacteriales: Atopobiaceae]), and negative loadings of genera previously reported to be propionate and acetate producing (*Bacteroides* and *Phascolarctobacterium* [Acidaminococcales: Acidaminococcaceae]). PC2 was thus referred to as the ‘butyrate-associated bacteria’ The propionate and acetate-associated bacteria (PC1) were unaffected by dietary fiber content (feeding treatment: P = 0.48, Table 3; Fig. 2C) whereas the butyrate-associated bacteria (PC2) increased with dietary fiber content (feeding treatment: P < 0.001, Table 3; Fig. 2D). The PCA biplot indicated directional alignment of all of the butyrate-associated bacteria (Ruminococcaceae UCG-014, *Prevotella, Ruminococcus torques* group, and *Olsenella*, 4 of 4 genera) with 9% dietary fiber samples, however, samples from the three different feeding treatments did not form distinct clusters (Fig. 2E).

### Insoluble fiber altered community structure in the mucosa

**Fig. 3:**
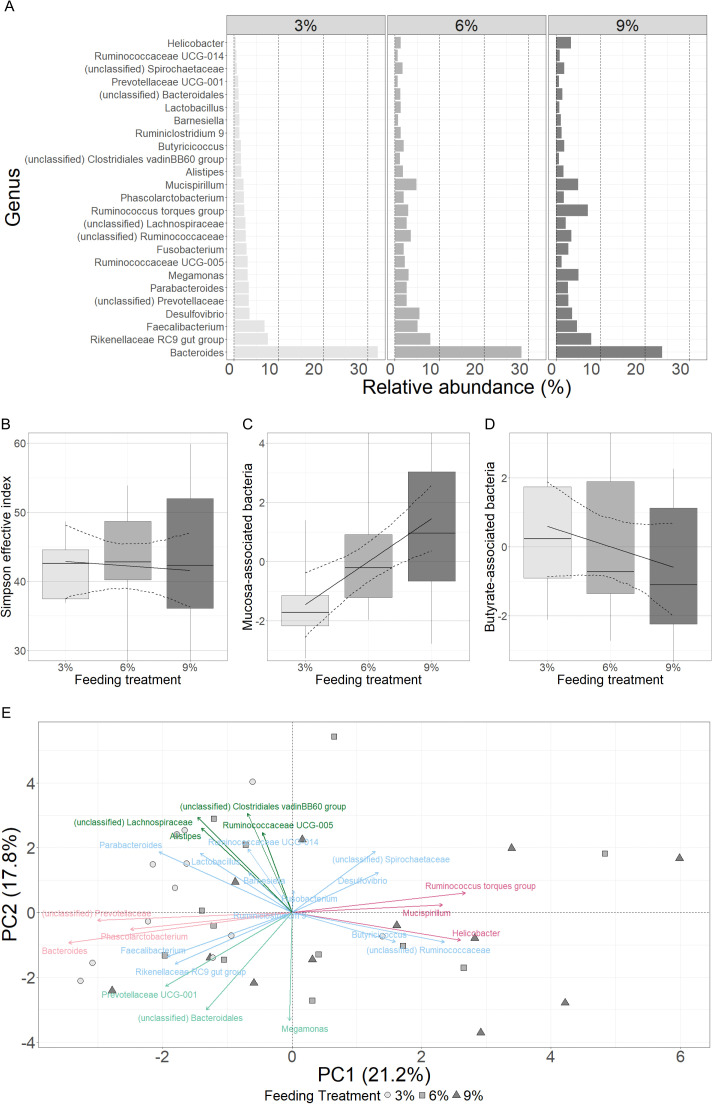
Microbial composition of the cecal mucosa of Lohmann Tradition hens fed three experimental diets that different in the amount of dietary fiber (3%, 6%, 9%) but not in metabolizable energy: (A) Relative abundance (%) of bacterial genera are presented as bar plots. Each bar represents the mean relative abundance of the respective genus for each feeding treatment. (B) Microbial diversity (Simpson effective index), (C) mucosa-associated bacteria, and (D) butyrate-associated bacteria, as an outcome of feeding treatment, are presented as box-and-whiskers plots. Each box represents the interquartile range, the thick line within the box indicates the median and the whiskers represent the range of the data. The solid lines indicate the model estimates and the dotted lines represent 95% confidence intervals. (E) Principal Component Analysis (PCA) biplot of CLR-transformed genus-level microbial relative abundance. Points represent individual samples by feeding treatment (circle: 3%; square: 6%; triangle: 9% dietary fiber), while arrows represent genera contributing to variation among samples. The first two principal components (PC) explained 21.2% and 17.8% of total variance respectively. The genera that loaded (loading threshold = ± 0.25) positively and negatively on PC1 are colored dark pink and light pink respectively; genera that loaded positively and negatively on PC2 are colored dark green and light green respectively; genera that did not load on either PC are colored blue.

Similar to the lumen, the mucosa was seen to be dominated by Bacteroidetes with ∼50% of all bacteria, followed by Firmicutes (∼35%) for each of the three feeding treatments. We observed 25 different bacterial genera in the mucosa as well, out of which 22 were common to the luminal samples (Fig. 3A).

The Simpson effective index of mucosal samples of the cecum was unaffected by dietary fiber content (feeding treatment: P = 0.742, Table 3; Fig. 3B).

Here as well, a PCA was performed to reduce dimensionality. Similar to the luminal samples, the first two PC in the mucosal samples were most strongly indicated by the PCA (Supplementary Figure 2). PC1 (21.2% of variance explained) was associated with positive loadings of genera previously reported to colonize the intestinal mucosa (members of the *R. torques* group, *Helicobacter* [Campylobacterales: Helicobacteraceae] and *Mucispirillum* [Deferrebacterales: Deferrebacteraceae]), and negative loadings of genera previously linked to fiber degradation (*Bacteroides*, unclassified Prevotellaceae, and *Phascolarctobacterium*). PC1 was thus referred to as the ‘mucosa-associated bacteria’. PC2 (17.8% of variance explained) was associated with positive loadings of genera reported as butyrate producing (unclassified genus from Clostridiales vadin BB60 family, unclassified Lachnospiraceae, and Ruminococcaceae UCG-005), interestingly the same three of the four genera that loaded negatively on PC1 in the luminal samples, and negative loadings of genera previously described as propionate and acetate producing (*Megamonas* [Selenomonadales: Selenomonadaceae], unclassified Bacteroidales, and Prevotellaceae UCG-001). PC2 was thus referred to as the ‘butyrate-associated bacteria’. The mucosa-associated bacteria (PC1) increased with dietary fiber content (feeding treatment: P = 0.002, Table 3; Fig. 3C). The butyrate-associated bacteria (PC2), on the other hand, remained unaffected by dietary fiber content (feeding treatment: P = 0.256, Table 3; Fig. 3D). The PCA biplot indicated limited directional alignment of a few of the mucosa-associated bacteria (*Helicobacter*, 1 of 3 genera) with the majority of the 9% dietary fiber samples, and no distinct clustering of samples according to the three feeding treatments (Fig. 3E).

## Discussion

**Fig. 4:**
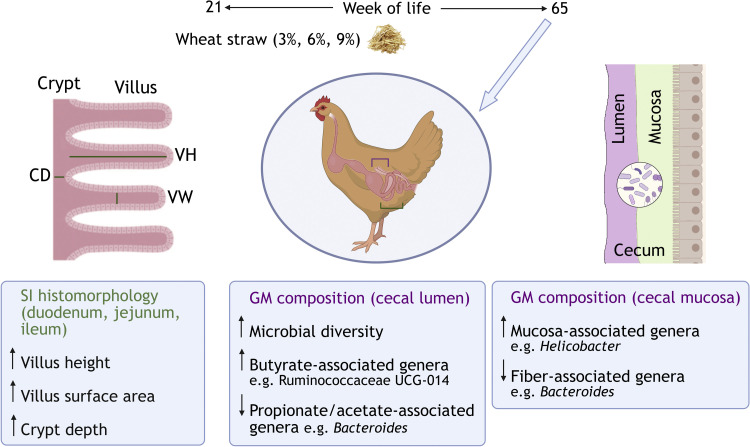
An overview of the results of increasing insoluble fiber (= 3%, 6%, 9% wheat straw) supplementation in the diet on small intestinal histomorphology and cecal microbial composition of Lohmann Tradition hens. Abbreviation: SI, small intestine; VH, villus height; VW, villus width; CD, crypt depth; GM, gut microbiota. Created in BioRender. Das, S. (2026) https://BioRender.com/iw3z4ob.

Dietary fiber is an essential component of poultry diets. Previous studies indicate that different sources of fiber may affect intestinal morphology and microbial composition in poultry, to varying extents, depending on properties such as solubility and fermentability (Jha and Mishra, 2021). The present study used increasing concentrations (3%, 6%, 9%) of ground wheat straw, as a source of insoluble fiber, in laying hen diets to investigate small intestinal histomorphology and cecal microbial composition. Unlike many studies in poultry that used insoluble fiber as nutrient diluents, the present study used isocaloric diets that differed in the relative amounts of the different energy sources. An overview of the results of the study is presented in Fig. 4.

### Histomorphology of the small intestine

Our results showed that villus height and villus surface area increased with increasing dietary fiber content, indicating a potential for increased nutrient absorption in the small intestine, because nutrients are absorbed through the villus surface (Tarachai and Yamauchi, 2000; Ensari and Marsh, 2018). The increase was more pronounced in the duodenum as compared to the jejunum and the ileum, which is consistent with the duodenum being a major site of nutrient absorption in chickens (van der Klis et al., 1990; Yamauchi, 2002) and other animals (e.g. humans: Kiela and Ghishan, 2016). We also observed an increase in crypt depth with increasing dietary fiber content in all three intestinal sections. As crypts are a principal site of epithelial cell proliferation in chickens (Uni et al., 1998), deeper crypts indicate faster cell turnover to support villus maintenance and renewal, reflected in the observed increase in villus height.

Consistent with our findings, small intestinal villus height or villus surface area or both increased with insoluble fiber supplementation using lignocellulose from week 1 to 4 in broilers (Makivic et al., 2019), using wheat bran from week 90 to 99 in laying hens (Abdollahi et al., 2021), and using sunflower meal from week 21 to 44 in laying hens (Koçer et al., 2021). These effects were observed at 3 to 4% crude fiber but higher concentrations, 3.5% (Abdollahi et al., 2021) or 5% (Koçer et al., 2021) did not improve villus architecture further. The authors hypothesized that an excess of fiber might be damaging to the intestinal mucosa. However, in the present study, we observed improvements in villus architecture when the hens were fed up to 9% fiber. This might be owing to our longer duration (week 21 to 65) of fiber feeding, potentially allowing enough time for the intestinal morphological adaptations in response to high dietary fiber. This is supported by Sklan et al. (2003) who found an increase in jejunal villus surface area when turkeys were fed up to 9% insoluble fiber (sunflower meal and soybean hulls) for a longer duration (week 1 to 14), but only up to 6% fiber when fed for a shorter duration (week 1 to 4). Moreover, in contrast to the above studies using mashed feed that might allow birds to selectively avoid the fibrous parts of the feed, we used pelleted feed to rule out such possibilities. Therefore, we argue that an individual or a combined effect of factors such as duration of feeding treatment, source of fiber used, form of feed and the age of tested birds might explain why we saw a continuous improvement (up to 9% fiber) in small intestinal histomorphology in the present study.

VH:CD is a useful criterion for estimating the nutrient absorptive capacity of the small intestine, as it reflects the balance between absorptive surface area (villus height) and epithelial cell turnover (crypt depth) (Montagne et al., 2003). For instance, Shang et al. (2020) reported that 6% wheat bran supplementation in pig diets increased VH:CD compared to a non-supplemented diet, even though changes in villus height and crypt depth were not statistically supported (Shang et al., 2020). This shows that the ratio, in some cases, might be more informative than villus height and crypt depth alone. However, we did not observe any effect of dietary fiber on VH:CD potentially owing to the simultaneous and proportional increase in both villus height and crypt depth with increasing fiber.

### Cecal microbial composition

Irrespective of the feeding treatment, Bacteroidetes was the most abundant phylum followed by Firmicutes in both lumen and mucosa of the cecum of 65-week-old laying hens. This is in line with the observation in laying hens that Bacteroidetes abundance gradually increased with age, accompanied by a simultaneous decrease in the abundance of Firmicutes (Videnska et al., 2014). Our findings demonstrated altered community structure at the genus level in both cecal lumen and mucosa in response to fiber supplementation in the diet, as indicated by changes in principal components derived from PCA. However, it is important to note that 16S rRNA gene sequencing provides limited functional information, and the metabolic roles of the observed genera cannot be confirmed in the present study. We therefore discuss the potential functional characteristics of these genera based on existing evidence from past studies.

In the cecal lumen, the butyrate-associated bacteria (PC2) increased with dietary fiber content. Several genera that contributed to PC2 have been previously associated with fiber fermentation and SCFA production. For instance, members of the Ruminococcaceae family, including the genus Ruminococcaceae UCG-014, have been reported as butyrate producers (Fusco et al., 2023). In addition, *R. torques* group, *Prevotella* and *Olsenella* are not butyrate producers themselves but have the potential to stimulate butyrate synthesis via cross-feeding. For instance, Berkhout et al. (2024) proposed that butyrate synthesis is stimulated in the presence of the mucin degrading *R. torques* (first described by Holdemann and Moore) as butyrate producers in the gut cross-feed on mucin degradation products. Similarly, the major fermentation products of *Prevotella* and *Olsenella*, i.e. succinate and lactate, respectively, can be utilized by butyrate producers to produce butyrate (Louis and Flint, 2017). Therefore, our findings suggest that increasing concentrations of dietary fiber has the potential to increase the relative abundance of butyrate-associated bacteria in the cecal lumen. This was supported by the alignment of the observed genera with samples in the PCA biplot, where the butyrate-associated bacteria were oriented towards the 9% dietary fiber samples and away from the 3% and 6% dietary fiber samples. Consistent with our observation, 2 to 6% insoluble fiber (e.g. lignocellulose, wheat bran) supplementation increased cecal microbial diversity and the relative abundances of butyrate producing bacteria in dual purpose chickens (Hou et al., 2020) and other farm animals (e.g. pigs: Shang et al., 2020).

With increasing fiber content, we also observed an increase in microbial diversity in the lumen. A highly diverse gut microbiota has been suggested to be indicative of a healthy gut (chickens: Yue et al., 2024; monogastric animals: Jha et al., 2019). Although the underlying mechanisms are still not completely understood, SCFA appeared as a mediator (Kumar et al., 2025). Western diets high in fat and low in fiber have been linked to a reduction in SCFA-producing bacteria with an associated decrease in fecal microbial diversity (humans: De Filippo et al., 2010). Although our 9% diet is comparable to a high fat diet in terms of the main energy source (Table 1), we observed an increase in luminal microbial diversity and butyrate-associated bacteria with increasing fiber content. Therefore, we assume that the potential negative effects associated with high levels of dietary fat were mitigated by the positive effects of high insoluble fiber in the present study.

To the best of our knowledge, this is the first study that investigated the effect of fiber supplementation on the mucosal microbial composition in laying hen cecum. We observed an increase in the mucosa-associated bacteria (PC1) with increasing dietary fiber content. The bacteria that contributed to PC1 included the *R. torques* group that has been reported as mucus degraders (Schaus et al., 2024), and the genera *Helicobacter* and *Mucispirillum* that are known to colonize the intestinal mucosa. In line with this observation, Kuffa et al. (2023) reported that a fiber-deficient diet led to a reduced relative abundance of *Mucispirillum* in the large intestinal mucosa of mice. Furthermore, the authors found that *Mucispirillum* is not a primary degrader of mucin but utilizes the end products of mucin degradation by *R. torques. Helicobacter* is a potentially pathogenic bacterial genus that colonizes the intestinal mucosa in broilers (Ceelen et al., 2007) and other animals (e.g. humans: Varon et al., 2009). *Helicobacter pullorum* (first described by Stanley et al.) has been reported in association with vibrionic hepatitis in laying hens (Stanley et al., 1994), a condition historically described as being characterized by diarrhea, weight loss and increased mortality in adult hens (Whenham et al., 1961). However, no clinical signs consistent with vibrionic hepatitis were observed in the present study, which is in agreement with findings from other studies in laying hens where *H. pullorum* was detected without overt disease (Javed et al., 2017). Therefore, we may argue that increasing dietary fiber content has the potential to increase the relative abundance of mucosa-associated bacteria in the cecal mucosa. This was reflected in the PCA biplot by a limited directional alignment of some of the mucosa-associated bacteria with the majority of the 9% dietary fiber samples, and opposing orientation of the 3% dietary fiber samples.

Although dietary carbohydrates are the main source of nutrients for gut microbiota, the mucus layer is an alternative source of host-derived glycans. In mice, a lack of dietary fiber has been shown to enhance degradation of the mucus layer (Desai et al., 2016; Schroeder, 2019). Accordingly, a high fiber diet may be associated with a more intact mucus layer that supports colonization by mucosa-associated bacteria, which may explain the observed increase in these genera with increasing fiber in our study.

### Association between small intestinal histomorphology and performance

Data on feed intake and performance traits of the hens used in this study, i.e. productive egg laying performance, body weight, and estimated feed conversion ratio, were published earlier by our research group (Patt et al., 2022). We observed that none of the performance traits differed between hens of the three feeding treatments, despite the potential for increased nutrient absorption in hens fed high fiber. In agreement with our observations, 3% wheat bran supplementation in 90-week-old laying hens increased jejunal villus height and villus surface area with no effects on laying performance (Abdollahi et al., 2021). However, contrary to this, Koçer et al. (2021) reported improvements in laying performance, body weight, and feed conversion ratio, but comparable feed intake, when hens were fed 4% (supplemented with sunflower meal) as compared to 3% crude fiber in the diet, alongside increased ileal villus height. In our study, a decline in estimated feed intake of the hens with increasing levels of fiber in the diet was observed (Patt et al., 2022). Therefore, it is plausible to speculate that feed intake and intestinal surface area might have balanced each other out, resulting in the comparable performance traits reported in Patt et al. (2022).

### Association between small intestinal histomorphology and cecal microbial composition: potential future directions

In the present study, a high dietary fiber content in laying hens seemed to have the potential to increase the relative abundance of butyrate-associated bacteria in the cecal lumen, associated with an improved small intestinal histomorphology. Similar associations between cecal abundance of butyrate-associated bacteria/potential butyrate producers, such as Lachnospiraceae and Ruminococcaceae, and increased villus height in the ileum have been reported in broilers (De Maesschalck et al., 2015). Although the underlying mechanism was not addressed by the present study, a potential pathway involves glucagon-like peptide 2 (**GLP-2**), which exerts intestinotrophic effects in broilers (Hu et al., 2010) and other species (rodents: Ramsanahie et al., 2004; pigs: Burrin et al., 2005) and is stimulated by butyrate (pigs, rodents, and humans: Tappenden et al., 2003). It therefore seems plausible to assume that the improved small intestinal histomorphology in response to high dietary fiber content is mediated by butyrate-induced GLP-2 activity, a potential direction for further investigation in chickens.

## Conclusion

The present study showed that insoluble fiber (i.e. wheat straw) supplementation using isocaloric diets improved the small intestinal histomorphology of laying hens in terms of enhanced villus height and villus surface area. Moreover, high fiber feeding increased microbial diversity in the cecal lumen and shifted the overall microbial community structure in both lumen and mucosa. Our findings indicated that increased fiber content has the potential to increase butyrate-associated bacteria in the lumen and mucosa-associated bacteria in the mucosa of the cecum. Therefore, we may conclude that feeding higher levels of insoluble fiber (i.e. up to 9%) over 45 weeks of life seemed to be beneficial to the intestinal health of the hens. These positive effects on gut health were observed with high fiber despite the high dietary fat content that is usually associated with compromised gut health. Therefore, higher levels of fiber in our diets mitigated the potential negative effects of high fat. The present study paves the way for future research investigating the functional characteristics of the detected genera to subsequently explore the underlying mechanisms behind the observed intestinal improvements owing to dietary insoluble fiber supplementation in laying hens.

## Funding

The study was funded by 10.13039/501100024886Friedrich-Loeffler-Institut, Federal Research Institute for Animal Health, Greifswald-Insel Riems, Germany.

## CRediT authorship contribution statement

**S. Das:** Writing – original draft, Visualization, Methodology, Formal analysis, Data curation, Writing – review & editing. **I. Halle:** Writing – review & editing, Project administration, Conceptualization. **T.M. Fuchs:** Writing – review & editing, Methodology. **A. Dudde:** Writing – review & editing, Investigation. **E.T. Krause:** Writing – review & editing, Investigation. **A. Patt:** Writing – review & editing, Supervision, Project administration, Methodology, Investigation, Data curation, Conceptualization.

## Disclosures

The authors declare that they have no known competing financial interests or personal relationships that could have appeared to influence the work reported in this paper.

## Appendix Supplementary materials

Image, application 1

## Footnotes

Supplementary material associated with this article can be found, in the online version, at doi:10.1016/j.psj.2026.107292.
