# Supplementary material for: Dietary fiber content influences small intestinal histomorphology and cecal microbial composition of laying hens
Source: Poult Sci. 2026 Jun 13;105(10):107292. doi: 10.1016/j.psj.2026.107292 (PMC13315197; doi:10.1016/j.psj.2026.107292)
Supplement: Supplementary file 1 [file mmc1.docx]

**Supplementary material to the article titled ‘Dietary fiber content influences small intestinal histomorphology and cecal microbial composition of laying hens’**

Table 1. Principal component analysis (PCA) on the relative luminal abundances of bacterial genera of Lohmann Tradition hens fed three experimental diets that differed in the amount of dietary fiber (3%, 6%, 9%) but not in metabolizable energy: proportion of variance, cumulative variance, and Eigenvalue of as well as genera loadings on the first nine principal components (PC) are presented. We interpreted the first two PC in the main article. Numbers in bold indicate loading of a genera on the first two PC for which the absolute value was ≥ 0.25 (threshold). We focused on interpreting loadings with absolute values ≥ 0.25 in the main article.

| Dataset PC no. | PC1^1^ | PC2^2^ | PC3 | PC4 | PC5 | PC6 | PC7 | PC8 | PC9 |
| --- | --- | --- | --- | --- | --- | --- | --- | --- | --- |
| Proportion of variance (%) | 27 | 15.6 | 10.2 | 7.5 | 6.2 | 5.4 | 4 | 4 | 3.5 |
| Cumulative variance (%) | 27 | 42.6 | 52.8 | 60.3 | 66.5 | 71.9 | 75.9 | 79.9 | 83.4 |
| Eigenvalue | 6.76 | 3.89 | 2.54 | 1.87 | 1.55 | 1.35 | 1.02 | 1 | 0.87 |
| *Alistipes* | −0.11 | 0.10 | −0.34 | 0.35 | −0.08 | 0.01 | 0.46 | −0.05 | −0.07 |
| *Bacteroides* | 0.09 | −**0.38** | −0.19 | −0.02 | −0.21 | −0.22 | −0.28 | −0.04 | 0.07 |
| *Barnesiella* | −0.09 | 0.13 | −0.01 | 0.27 | 0.54 | 0.23 | −0.05 | −0.16 | −0.19 |
| *Butyricicoccus* | −0.24 | 0.18 | 0.27 | −0.03 | −0.13 | −0.15 | −0.17 | −0.03 | 0.31 |
| *Desulfovibrio* | −0.01 | −0.15 | 0.21 | 0.42 | −0.07 | 0.16 | −0.16 | 0.44 | −0.34 |
| *Faecalibacterium* | 0.09 | 0.05 | 0.06 | −0.59 | 0.13 | 0.13 | 0.09 | −0.19 | −0.24 |
| *Fusobacterium* | −0.13 | −0.02 | 0.48 | 0.00 | 0.16 | 0.10 | 0.13 | −0.32 | 0.03 |
| *Lactobacillus* | −0.06 | 0.19 | −0.34 | −0.10 | −0.03 | −0.37 | −0.13 | −0.02 | −0.29 |
| *Megamonas* | 0.23 | 0.18 | 0.18 | −0.16 | −0.14 | 0.14 | −0.24 | 0.25 | 0.10 |
| *Olsenella* | 0.13 | **0.31** | −0.15 | −0.02 | 0.32 | 0.12 | −0.22 | 0.11 | −0.08 |
| *Parabacteroides* | −0.23 | −0.08 | −0.07 | −0.15 | −0.17 | 0.31 | 0.12 | 0.29 | −0.29 |
| *Phascolarctobacterium* | 0.07 | −**0.33** | −0.08 | 0.03 | −0.05 | 0.44 | −0.40 | −0.13 | 0.07 |
| *Prevotella* | 0.14 | **0.33** | 0.08 | 0.12 | −0.35 | 0.22 | 0.16 | 0.07 | 0.04 |
| Prevotellaceae UCG-001 | **0.31** | −0.09 | −0.17 | 0.06 | 0.13 | −0.06 | 0.00 | 0.11 | 0.07 |
| Rikenellaceae RC9 gut group | **0.30** | 0.12 | 0.02 | 0.10 | −0.16 | −0.03 | 0.00 | −0.19 | −0.16 |
| *Ruminiclostridium 9* | −**0.25** | 0.14 | −0.03 | 0.19 | −0.18 | 0.14 | −0.18 | −0.28 | 0.22 |
| Ruminococcaceae UCG-005 | −**0.29** | −0.21 | −0.13 | −0.04 | 0.18 | −0.07 | 0.07 | 0.00 | 0.11 |
| Ruminococcaceae UCG-014 | −0.15 | **0.31** | −0.12 | −0.10 | −0.23 | 0.22 | 0.11 | 0.06 | 0.13 |
| *Ruminococcus torques* group | −0.16 | **0.32** | −0.02 | −0.03 | 0.20 | −0.09 | −0.33 | 0.22 | −0.04 |
| Bacteroidales (unclassified) | **0.30** | 0.12 | 0.08 | −0.04 | −0.12 | −0.20 | 0.05 | −0.02 | −0.13 |
| Clostridiales vadin BB60 group (unclassified) | −**0.27** | −0.17 | −0.06 | −0.01 | −0.15 | −0.05 | −0.04 | −0.06 | −0.24 |
| Gastranaerophilales (unclassified) | −0.22 | −0.07 | −0.19 | −0.37 | −0.04 | 0.25 | 0.06 | 0.14 | −0.03 |
| Lachnospiraceae (unclassified) | −**0.31** | 0.17 | −0.11 | 0.01 | −0.01 | −0.16 | −0.20 | 0.12 | 0.16 |
| Prevotellaceae (unclassified) | 0.19 | −0.09 | −0.19 | −0.03 | 0.26 | 0.12 | 0.23 | 0.30 | 0.53 |
| Ruminococcaceae (unclassified) | −0.15 | −0.12 | 0.39 | −0.05 | 0.09 | −0.28 | 0.21 | 0.37 | −0.02 |

^1^Propionate and acetate-associated bacteria

^2^Butyrate-associated bacteria

Table 2. Principal component analysis (PCA) on the relative mucosal abundances of bacterial genera of Lohmann Tradition hens fed three experimental diets that differed in the amount of dietary fiber (3%, 6%, 9%) but not in metabolizable energy: proportion of variance, cumulative variance, and Eigenvalue of as well as genera loadings on the first nine principal components (PC) are presented. We interpreted the first two PC in the main article. Numbers in bold indicate loading of a genera on the first two PC for which the absolute value was ≥ 0.25 (threshold). We focused on interpreting loadings with absolute values ≥ 0.25 in the main article.

| Dataset PC no. | PC1^1^ | PC2^2^ | PC3 | PC4 | PC5 | PC6 | PC7 | PC8 | PC9 |
| --- | --- | --- | --- | --- | --- | --- | --- | --- | --- |
| Proportion of variance (%) | 21.2 | 17.8 | 11.2 | 8 | 7.2 | 5.3 | 3.9 | 3.3 | 3.3 |
| Cumulative variance (%) | 21.2 | 39 | 50.2 | 58.2 | 65.4 | 70.7 | 74.6 | 77.9 | 81.2 |
| Eigenvalue | 5.3 | 4.45 | 2.79 | 2 | 1.8 | 1.32 | 0.97 | 0.83 | 0.82 |
| *Alistipes* | −0.15 | **0.29** | 0.30 | −0.14 | 0.00 | 0.13 | −0.01 | −0.04 | 0.11 |
| *Bacteroides* | −**0.38** | −0.10 | −0.10 | −0.07 | −0.01 | 0.06 | −0.13 | 0.14 | 0.03 |
| *Barnesiella* | −0.07 | 0.14 | 0.05 | 0.15 | 0.46 | 0.26 | 0.15 | −0.28 | −0.37 |
| *Butyricicoccus* | 0.18 | −0.10 | −0.33 | −0.21 | 0.01 | −0.10 | −0.44 | −0.23 | −0.14 |
| *Desulfovibrio* | 0.15 | 0.14 | 0.09 | −0.31 | −0.34 | −0.09 | 0.37 | 0.04 | −0.26 |
| *Faecalibacterium* | −0.21 | −0.15 | −0.23 | 0.30 | −0.13 | −0.12 | −0.13 | −0.41 | −0.09 |
| *Fusobacterium* | 0.00 | 0.07 | −0.23 | 0.10 | 0.45 | −0.30 | 0.17 | 0.23 | 0.07 |
| *Helicobacter* | **0.29** | −0.09 | 0.23 | 0.01 | 0.23 | 0.06 | −0.05 | −0.28 | −0.08 |
| *Lactobacillus* | −0.16 | 0.20 | 0.10 | 0.07 | −0.44 | −0.12 | −0.09 | −0.17 | 0.25 |
| *Megamonas* | 0.00 | −**0.37** | 0.03 | −0.02 | 0.01 | −0.26 | −0.04 | 0.18 | −0.02 |
| *Mucispirillum* | **0.26** | 0.03 | 0.17 | −0.38 | 0.06 | −0.01 | −0.24 | −0.22 | 0.02 |
| *Parabacteroides* | −0.23 | 0.21 | −0.02 | −0.02 | −0.09 | −0.26 | −0.24 | 0.10 | −0.43 |
| *Phascolarctobacterium* | −**0.27** | −0.06 | −0.21 | −0.09 | 0.24 | −0.23 | 0.21 | −0.08 | 0.25 |
| Prevotellaceae UCG-001 | −0.22 | −**0.25** | 0.10 | −0.13 | −0.04 | 0.13 | 0.45 | −0.30 | −0.06 |
| Rikenellaceae RC9 gut group | −0.20 | −0.17 | 0.29 | −0.07 | 0.09 | 0.21 | −0.26 | 0.27 | −0.05 |
| *Ruminiclostridium* 9 | −0.01 | 0.02 | −0.15 | −0.49 | 0.19 | 0.16 | −0.08 | −0.04 | 0.45 |
| Ruminococcaceae UCG-005 | −0.05 | **0.27** | −0.36 | −0.08 | −0.09 | 0.31 | 0.06 | −0.14 | −0.11 |
| Ruminococcaceae UCG-014 | −0.07 | 0.22 | 0.31 | 0.09 | 0.22 | −0.17 | −0.21 | −0.23 | 0.03 |
| *Ruminococcus torques* group | **0.29** | 0.07 | −0.05 | 0.42 | −0.10 | 0.15 | −0.04 | 0.09 | 0.18 |
| Bacteroidales (unclassified) | −0.15 | −**0.33** | 0.15 | 0.05 | 0.00 | −0.05 | −0.20 | −0.16 | 0.00 |
| Clostridiales vadin BB60 group (unclassified) | −0.08 | **0.34** | −0.13 | 0.05 | 0.11 | 0.26 | −0.21 | 0.25 | −0.06 |
| Lachnospiraceae (unclassified) | −0.16 | **0.32** | −0.12 | −0.05 | 0.01 | −0.27 | 0.00 | −0.23 | 0.13 |
| Prevotellaceae (unclassified) | −**0.33** | −0.03 | 0.04 | −0.23 | 0.01 | 0.03 | −0.01 | 0.12 | −0.29 |
| Ruminococcaceae (unclassified) | 0.24 | −0.10 | −0.29 | −0.19 | 0.00 | −0.06 | 0.00 | 0.04 | −0.27 |
| Spirochaetaceae (unclassified) | 0.14 | 0.21 | 0.24 | −0.06 | 0.12 | −0.45 | 0.09 | 0.08 | −0.05 |

^1^Mucosa-associated bacteria

^2^Butyrate-associated bacteria


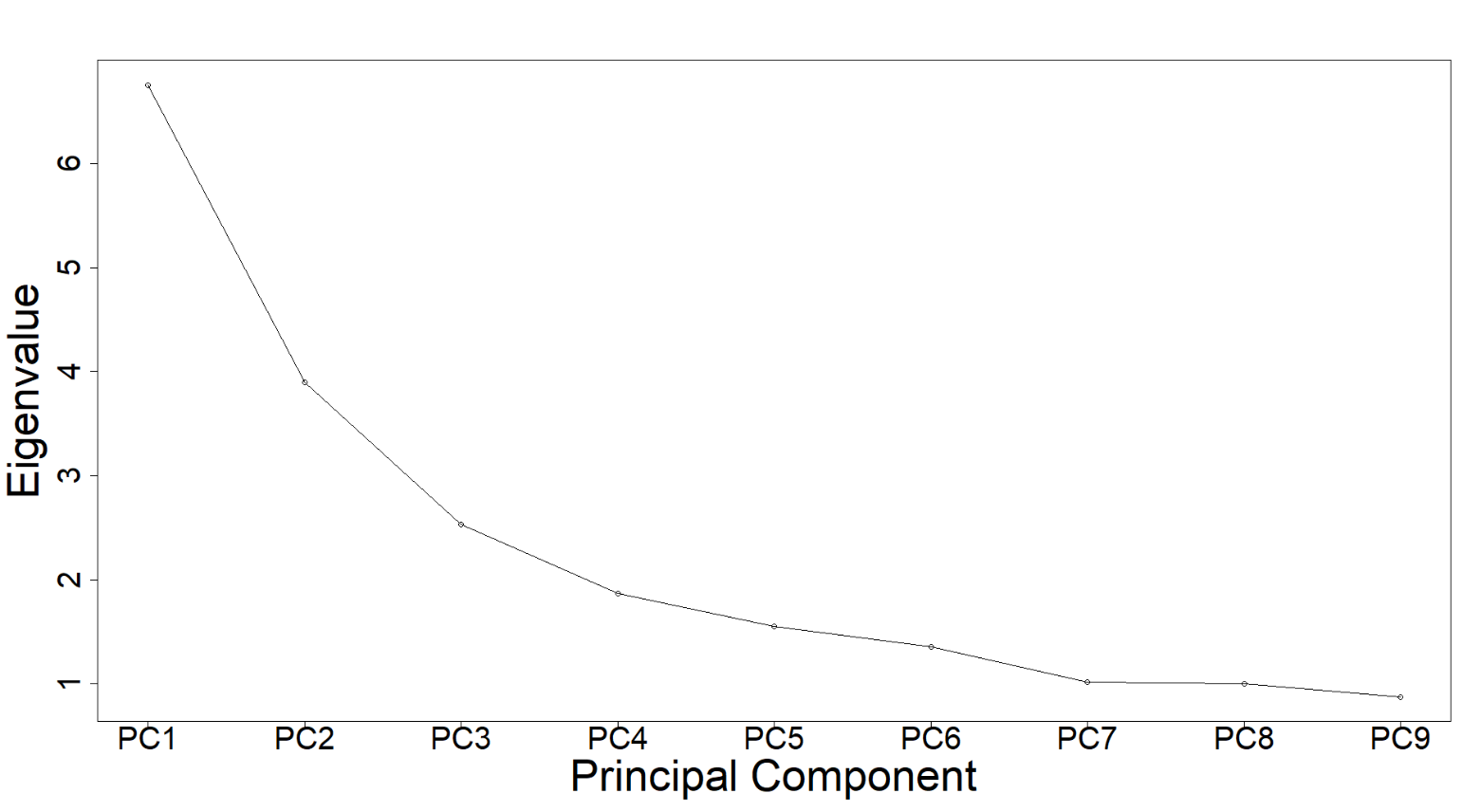


Figure 1. Scree plot of eigenvalues showing the variance explained by the first nine principal components (PC) from a principal component analysis (PCA) performed on the relative luminal abundances of bacterial genera of Lohmann Tradition hens fed three experimental diets that differed in the amount of dietary fiber (3%, 6%, 9%) but not in metabolizable energy. Each point on the curve represents a PC, with higher eigenvalues indicating that the component captures more variance in the dataset.


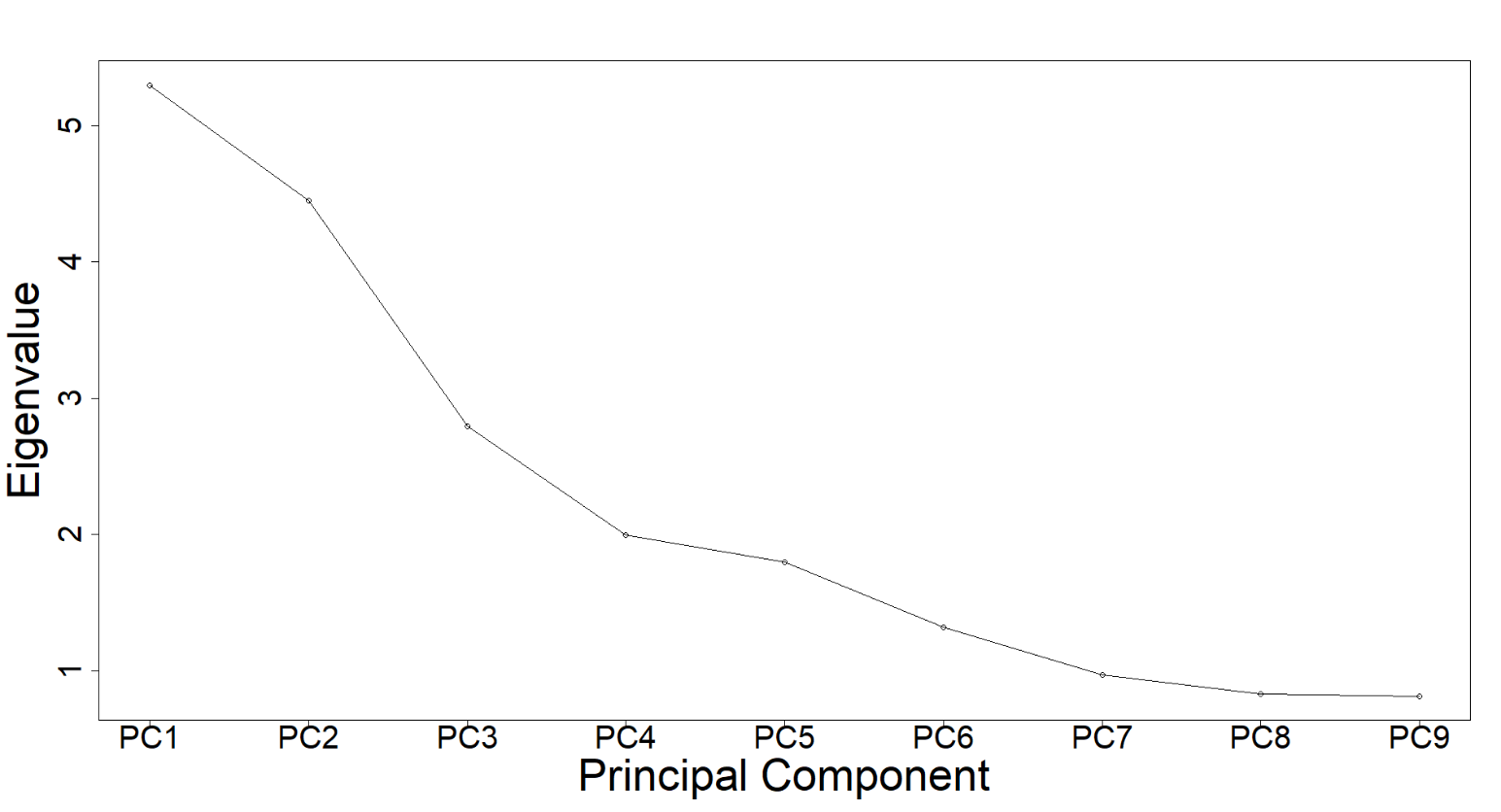


Figure 2. Scree plot of eigenvalues showing the variance explained by the first nine principal components (PC) from a principal component analysis (PCA) performed on the relative mucosal abundances of bacterial genera of Lohmann Tradition hens fed three experimental diets that differed in the amount of dietary fiber (3%, 6%, 9%) but not in metabolizable energy. Each point on the curve represents a PC, with higher eigenvalues indicating that the component captures more variance in the dataset.
